# Dimethyl 4-eth­oxy-1-(4-methyl-2-pyri­dyl)-5-oxo-2,5-dihydro-1*H*-pyrrole-2,3-dicarboxyl­ate

**DOI:** 10.1107/S1600536810012882

**Published:** 2010-04-17

**Authors:** Mohammad Anary-Abbasinejad, Marziyeh Mirhosseini, Masoumeh Tabatabaee

**Affiliations:** a Department of Chemistry, Islamic Azad University Yazd Branch, Yazd, Iran

## Abstract

In the title compound, C_16_H_18_N_2_O_6_, the dihedral angle between the aromatic ring planes is 8.11 (6)°. One of the O atoms is disordered over two sites of equal occupancy. In the crystal structure, aromatic π–π stacking [centroid-to-centroid separation = 3.5503 (8) Å] helps to consolidate the packing.

## Related literature

For background on 3-pyrrolines as synthetic inter­mediates, see: Tarnchompoo *et al.* (1987 ▶); Bienz *et al.* (1989 ▶). For further synthetic details, see: Anary-Abbasinejad *et al.* (2010 ▶).
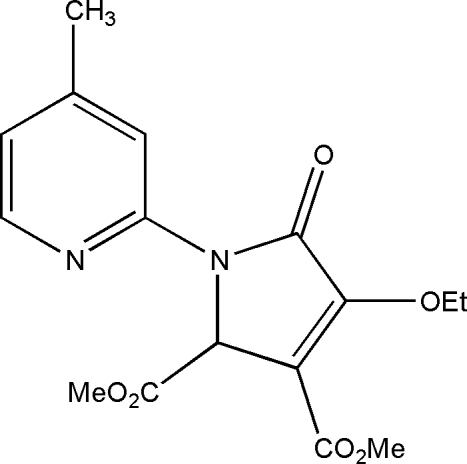


## Experimental

### 

#### Crystal data

C_16_H_18_N_2_O_6_
*M* *_r_* = 334.32
Triclinic, 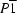

*a* = 9.2674 (6) Å
*b* = 9.4219 (6) Å
*c* = 10.7650 (7) Å
α = 87.692 (2)°
β = 72.037 (1)°
γ = 61.797 (1)°
*V* = 781.73 (9) Å^3^
*Z* = 2
Mo *K*α radiation
μ = 0.11 mm^−1^
*T* = 120 K
0.22 × 0.19 × 0.15 mm

#### Data collection

Bruker SMART 1000 CCD diffractometer
Absorption correction: multi-scan (*SADABS*; Bruker, 1998 ▶) *T*_min_ = 0.976, *T*_max_ = 0.984
14075 measured reflections
4340 independent reflections
3811 reflections with *I* > 2σ(*I*)
*R*_int_ = 0.020

#### Refinement

*R*[*F* ^2^ > 2σ(*F* ^2^)] = 0.043
*wR*(*F* ^2^) = 0.119
*S* = 1.00
4340 reflections
231 parameters
8 restraints
H-atom parameters constrained
Δρ_max_ = 0.37 e Å^−3^
Δρ_min_ = −0.29 e Å^−3^

### 

Data collection: *SMART* (Bruker, 1998 ▶); cell refinement: *SAINT-Plus* (Bruker, 1998 ▶); data reduction: *SAINT-Plus*; program(s) used to solve structure: *SHELXTL* (Sheldrick, 2008 ▶); program(s) used to refine structure: *SHELXTL*; molecular graphics: *SHELXTL*; software used to prepare material for publication: *SHELXTL*.

## Supplementary Material

Crystal structure: contains datablocks I, global. DOI: 10.1107/S1600536810012882/hb5395sup1.cif

Structure factors: contains datablocks I. DOI: 10.1107/S1600536810012882/hb5395Isup2.hkl

Additional supplementary materials:  crystallographic information; 3D view; checkCIF report

## Figures and Tables

**Fig. 1.**
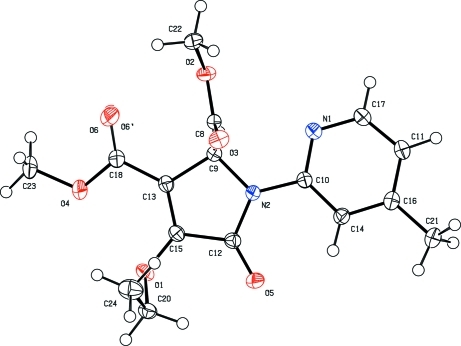
The molecular structure of (I). Atoms are represented by 50% displacement ellipsoids. The occupancies of positions of disordered O6 atom are equal (0.5).

**Fig. 2.**
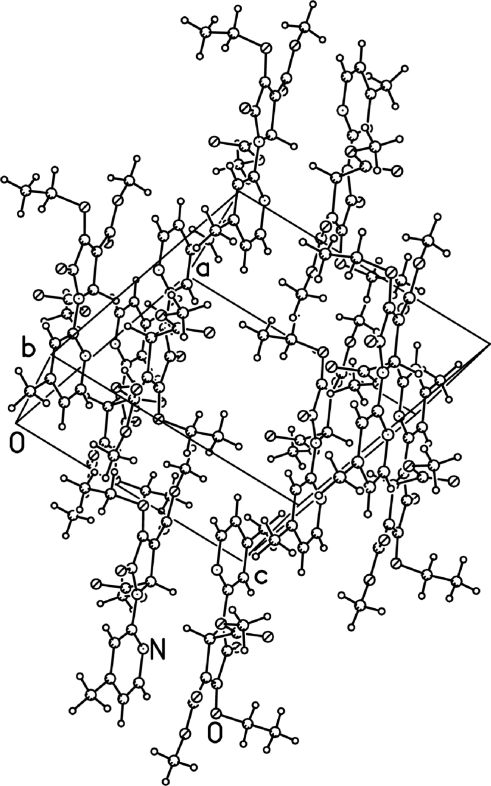
The overview of I crystal packing. Atoms are represented by spheres.

**Fig. 3.**
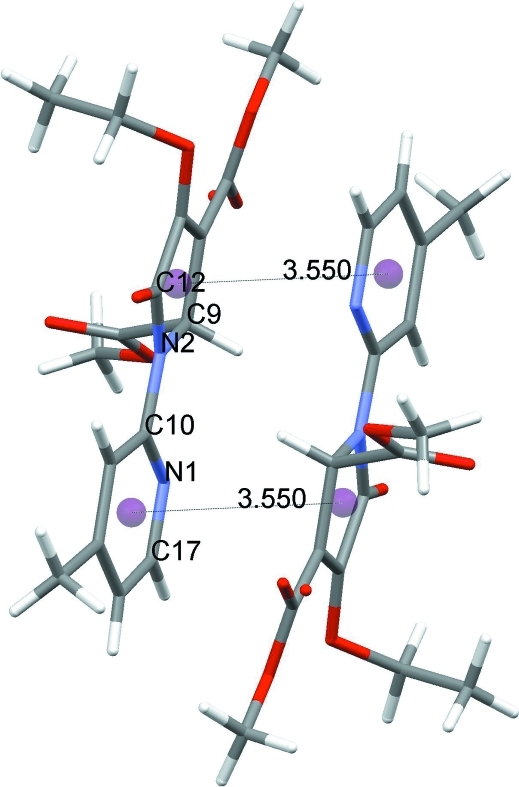
Representation of π–π stacking in I.

## supplementary crystallographic information

### Comment

(N-Substituted 3-pyrrolines serve as useful synthetic intermediates (Tarnchompoo *et al.*, 1987; Bienz *et al.*, 1989). Recently we reported a one-pot procedure for the synthesis of some dialkyl *N*-(3-methyl-2-pyridyl)-4-ethoxy-5-oxo-2,5-dihydro-1*H*- pyrole-2,3-dicarboxylate derivatives (Anary-Abbasinejad *et al.*, 2010).

Here we report the synthesis and crystal structure of the title compound, (I). It is rational to assume that compound 1 (Fig. 1) is produced from the initial production of ylide intermediate produced from three-component reaction of DMAD, 4-methyl-2-aminopyridine and triphenylphosphine, which then reacted with ethyl chlorooxoacetate to produce an oxamate derivative that underwent intramolecular Wittig reaction to give the products. Crystal packing of I is shown in Fig. 2. A considerable feature of the compound (I) is the presence of π–π stacking interactions between six and five membered rings (Fig. 3) with distance 3.5503 (8) Å for *Cg2*···*Cg3* (*Cg2* = N1/C10–C17 and *Cg3* = N2/C9–C12).

### Experimental

To a magnetically stirred solution of PPh_3_ (0.26 g, 1 mmol) and 4-methyl-2-aminopyridine (0.9 g, 1 mmol) in CH_2_Cl_2_(10 ml) was added drop-wise a mixture of dimethyl acetylenedicarboxylate DMAD (0.14 g, 1 mmol) in CH_2_Cl_2_ (3 ml) at room temperature over 2 min. The reaction mixture was then stirred for one more minute, then triethylamine (1 mmol) and ethyl chlorooxoacetate (1 mmol) was added and the reaction mixture was stirred for more 24 h. Solvent was evaporated and the residue was purified by column chromatography on SiO_2_ using EtOAC–hexane (1:4) mixture as eluent. The solid formed was filtrated, recrystallised from dichloromethane/ethanol (2:1) to yield colourless prisms of (I).

### Refinement

The H(C) atoms were placed in calculated positions and refined as riding with U_iso_(H) = 1.2U_eq_(C).

### Crystal data

**Table:**

C_16_H_18_N_2_O_6_	*Z* = 2
*M**_r_* = 334.32	*F*(000) = 352
Triclinic, *P*1	*D*_x_ = 1.420 Mg m^−^^3^
Hall symbol: -P 1	Mo *K*α radiation, λ = 0.71073 Å
*a* = 9.2674 (6) Å	Cell parameters from 259 reflections
*b* = 9.4219 (6) Å	θ = 3–29°
*c* = 10.7650 (7) Å	µ = 0.11 mm^−^^1^
α = 87.692 (2)°	*T* = 120 K
β = 72.037 (1)°	Prism, colourless
γ = 61.797 (1)°	0.22 × 0.19 × 0.15 mm
*V* = 781.73 (9) Å^3^	

### Data collection

**Table:**

Bruker SMART 1000 CCD diffractometer	4340 independent reflections
Radiation source: fine-focus sealed tube	3811 reflections with *I* > 2σ(*I*)
graphite	*R*_int_ = 0.020
ω scans	θ_max_ = 29.5°, θ_min_ = 2.0°
Absorption correction: multi-scan (*SADABS*; Bruker, 1998)	*h* = −12→12
*T*_min_ = 0.976, *T*_max_ = 0.984	*k* = −13→13
14075 measured reflections	*l* = −14→14

### Refinement

**Table:**

Refinement on *F*^2^	Primary atom site location: structure-invariant direct methods
Least-squares matrix: full	Secondary atom site location: difference Fourier map
*R*[*F*^2^ > 2σ(*F*^2^)] = 0.043	Hydrogen site location: inferred from neighbouring sites
*wR*(*F*^2^) = 0.119	H-atom parameters constrained
*S* = 1.00	*w* = 1/[σ^2^(*F*_o_^2^) + (0.0685*P*)^2^ + 0.3063*P*] where *P* = (*F*_o_^2^ + 2*F*_c_^2^)/3
4340 reflections	(Δ/σ)_max_ = 0.001
231 parameters	Δρ_max_ = 0.37 e Å^−^^3^
8 restraints	Δρ_min_ = −0.29 e Å^−^^3^

### Special details

**Table:**

Geometry. All esds (except the esd in the dihedral angle between two l.s. planes) are estimated using the full covariance matrix. The cell esds are taken into account individually in the estimation of esds in distances, angles and torsion angles; correlations between esds in cell parameters are only used when they are defined by crystal symmetry. An approximate (isotropic) treatment of cell esds is used for estimating esds involving l.s. planes.
Refinement. Refinement of *F*^2^ against ALL reflections. The weighted *R*-factor wR and goodness of fit *S* are based on *F*^2^, conventional *R*-factors *R* are based on *F*, with *F* set to zero for negative *F*^2^. The threshold expression of *F*^2^ > σ(*F*^2^) is used only for calculating *R*-factors(gt) etc. and is not relevant to the choice of reflections for refinement. *R*-factors based on *F*^2^ are statistically about twice as large as those based on *F*, and *R*- factors based on ALL data will be even larger.

### Fractional atomic coordinates and isotropic or equivalent isotropic displacement parameters (Å^2^)

**Table:**

	*x*	*y*	*z*	*U*_iso_*/*U*_eq_	Occ. (<1)
O1	0.17590 (10)	0.52219 (10)	0.39546 (8)	0.02303 (17)	
O2	0.40451 (10)	0.95161 (9)	0.14840 (8)	0.02268 (17)	
O3	0.51098 (11)	0.79492 (10)	0.29618 (8)	0.02475 (18)	
N1	0.71510 (11)	0.58162 (11)	−0.02268 (9)	0.01973 (18)	
O4	−0.06090 (10)	0.84294 (10)	0.37603 (9)	0.02895 (19)	
O5	0.54578 (10)	0.30748 (9)	0.25074 (8)	0.02478 (18)	
N2	0.52930 (11)	0.53670 (10)	0.15196 (8)	0.01807 (17)	
C8	0.44689 (12)	0.82101 (12)	0.21038 (10)	0.01806 (19)	
C9	0.39637 (12)	0.70530 (12)	0.16169 (10)	0.01788 (19)	
H9A	0.3761	0.7310	0.0756	0.021*	
C10	0.69758 (12)	0.47752 (12)	0.06007 (9)	0.01754 (19)	
C11	1.01376 (13)	0.37860 (13)	−0.12410 (10)	0.0217 (2)	
H11A	1.1218	0.3474	−0.1911	0.026*	
C12	0.46748 (13)	0.45026 (12)	0.23786 (10)	0.01882 (19)	
C13	0.23818 (13)	0.71222 (12)	0.26432 (10)	0.01865 (19)	
C14	0.83205 (13)	0.32265 (12)	0.05951 (10)	0.0201 (2)	
H14A	0.8133	0.2538	0.1220	0.024*	
C15	0.28055 (13)	0.56764 (12)	0.30958 (10)	0.0190 (2)	
C16	0.99386 (13)	0.27226 (13)	−0.03493 (10)	0.0209 (2)	
C17	0.87312 (13)	0.53027 (13)	−0.11305 (10)	0.0216 (2)	
H17A	0.8889	0.6027	−0.1729	0.026*	
C18	0.07123 (14)	0.86461 (13)	0.30661 (11)	0.0230 (2)	
C20	0.23239 (15)	0.44492 (14)	0.50530 (11)	0.0242 (2)	
H20A	0.3508	0.3514	0.4706	0.029*	
H20B	0.1538	0.4040	0.5559	0.029*	
C21	1.14368 (15)	0.10896 (14)	−0.03822 (13)	0.0295 (2)	
H21A	1.2431	0.1216	−0.0393	0.044*	
H21B	1.1749	0.0388	−0.1175	0.044*	
H21C	1.1108	0.0598	0.0400	0.044*	
C22	0.40700 (15)	1.08725 (13)	0.20518 (12)	0.0263 (2)	
H22A	0.4173	1.1584	0.1380	0.039*	
H22B	0.5061	1.0464	0.2372	0.039*	
H22C	0.2993	1.1485	0.2787	0.039*	
C23	−0.22672 (15)	0.98915 (16)	0.42475 (13)	0.0347 (3)	
H23A	−0.3177	0.9600	0.4659	0.052*	
H23B	−0.2518	1.0504	0.3514	0.052*	
H23C	−0.2230	1.0560	0.4899	0.052*	
C24	0.23145 (19)	0.56449 (17)	0.59437 (13)	0.0351 (3)	
H24A	0.2595	0.5142	0.6712	0.053*	
H24B	0.1163	0.6606	0.6235	0.053*	
H24C	0.3179	0.5966	0.5465	0.053*	
O6'	0.0517 (16)	0.9913 (13)	0.2663 (9)	0.0417 (17)	0.50
O6	0.0634 (17)	0.9971 (12)	0.2975 (10)	0.0393 (15)	0.50

### Atomic displacement parameters (Å^2^)

**Table:**

	*U*^11^	*U*^22^	*U*^33^	*U*^12^	*U*^13^	*U*^23^
O1	0.0207 (4)	0.0295 (4)	0.0238 (4)	−0.0162 (3)	−0.0077 (3)	0.0093 (3)
O2	0.0264 (4)	0.0174 (3)	0.0276 (4)	−0.0124 (3)	−0.0107 (3)	0.0065 (3)
O3	0.0280 (4)	0.0229 (4)	0.0262 (4)	−0.0124 (3)	−0.0126 (3)	0.0039 (3)
N1	0.0194 (4)	0.0210 (4)	0.0196 (4)	−0.0107 (3)	−0.0058 (3)	0.0030 (3)
O4	0.0168 (4)	0.0254 (4)	0.0354 (4)	−0.0078 (3)	−0.0006 (3)	0.0022 (3)
O5	0.0237 (4)	0.0182 (4)	0.0318 (4)	−0.0108 (3)	−0.0076 (3)	0.0060 (3)
N2	0.0161 (4)	0.0154 (4)	0.0202 (4)	−0.0072 (3)	−0.0033 (3)	0.0018 (3)
C8	0.0155 (4)	0.0157 (4)	0.0198 (4)	−0.0070 (3)	−0.0028 (3)	0.0017 (3)
C9	0.0168 (4)	0.0166 (4)	0.0192 (4)	−0.0078 (4)	−0.0049 (3)	0.0024 (3)
C10	0.0175 (4)	0.0184 (4)	0.0175 (4)	−0.0097 (4)	−0.0048 (3)	0.0001 (3)
C11	0.0188 (4)	0.0264 (5)	0.0197 (4)	−0.0118 (4)	−0.0044 (3)	0.0003 (4)
C12	0.0195 (4)	0.0188 (4)	0.0204 (4)	−0.0111 (4)	−0.0063 (3)	0.0024 (3)
C13	0.0168 (4)	0.0204 (5)	0.0190 (4)	−0.0096 (4)	−0.0051 (3)	0.0017 (3)
C14	0.0192 (4)	0.0181 (4)	0.0217 (5)	−0.0086 (4)	−0.0059 (4)	0.0015 (3)
C15	0.0181 (4)	0.0213 (5)	0.0202 (4)	−0.0118 (4)	−0.0059 (3)	0.0030 (3)
C16	0.0183 (4)	0.0208 (5)	0.0223 (5)	−0.0082 (4)	−0.0065 (4)	−0.0008 (4)
C17	0.0215 (5)	0.0250 (5)	0.0196 (4)	−0.0128 (4)	−0.0061 (4)	0.0037 (4)
C18	0.0197 (5)	0.0227 (5)	0.0223 (5)	−0.0085 (4)	−0.0044 (4)	0.0024 (4)
C20	0.0246 (5)	0.0249 (5)	0.0242 (5)	−0.0133 (4)	−0.0081 (4)	0.0090 (4)
C21	0.0199 (5)	0.0238 (5)	0.0350 (6)	−0.0051 (4)	−0.0052 (4)	0.0027 (4)
C22	0.0280 (5)	0.0168 (5)	0.0329 (6)	−0.0120 (4)	−0.0065 (4)	0.0016 (4)
C23	0.0176 (5)	0.0322 (6)	0.0358 (6)	−0.0034 (5)	0.0009 (4)	0.0014 (5)
C24	0.0472 (7)	0.0378 (7)	0.0276 (6)	−0.0245 (6)	−0.0155 (5)	0.0082 (5)
O6'	0.026 (2)	0.026 (2)	0.051 (4)	−0.0063 (13)	0.004 (2)	0.014 (2)
O6	0.0263 (16)	0.0202 (13)	0.058 (4)	−0.0087 (11)	−0.002 (3)	0.004 (2)

### Geometric parameters (Å, °)

**Table:**

O1—C15	1.3364 (12)	C13—C18	1.4769 (14)
O1—C20	1.4679 (13)	C14—C16	1.3904 (14)
O2—C8	1.3238 (12)	C14—H14A	0.9500
O2—C22	1.4515 (13)	C16—C21	1.5004 (15)
O3—C8	1.2035 (13)	C17—H17A	0.9500
N1—C10	1.3315 (13)	C18—O6'	1.204 (10)
N1—C17	1.3471 (13)	C18—O6	1.218 (10)
O4—C18	1.3263 (13)	C20—C24	1.5037 (17)
O4—C23	1.4472 (14)	C20—H20A	0.9900
O5—C12	1.2165 (13)	C20—H20B	0.9900
N2—C12	1.3785 (12)	C21—H21A	0.9800
N2—C10	1.4115 (12)	C21—H21B	0.9800
N2—C9	1.4615 (12)	C21—H21C	0.9800
C8—C9	1.5369 (14)	C22—H22A	0.9800
C9—C13	1.5134 (13)	C22—H22B	0.9800
C9—H9A	1.0000	C22—H22C	0.9800
C10—C14	1.3999 (14)	C23—H23A	0.9800
C11—C17	1.3830 (15)	C23—H23B	0.9800
C11—C16	1.3954 (15)	C23—H23C	0.9800
C11—H11A	0.9500	C24—H24A	0.9800
C12—C15	1.5011 (14)	C24—H24B	0.9800
C13—C15	1.3440 (14)	C24—H24C	0.9800
			
C15—O1—C20	116.43 (8)	N1—C17—H17A	118.0
C8—O2—C22	116.22 (9)	C11—C17—H17A	118.0
C10—N1—C17	116.47 (9)	O6'—C18—O4	122.5 (6)
C18—O4—C23	115.57 (9)	O6—C18—O4	123.7 (6)
C12—N2—C10	127.04 (9)	O6'—C18—C13	123.4 (6)
C12—N2—C9	112.20 (8)	O6—C18—C13	121.9 (6)
C10—N2—C9	120.74 (8)	O4—C18—C13	113.18 (9)
O3—C8—O2	126.28 (10)	O1—C20—C24	110.36 (9)
O3—C8—C9	123.12 (9)	O1—C20—H20A	109.6
O2—C8—C9	110.56 (8)	C24—C20—H20A	109.6
N2—C9—C13	102.82 (8)	O1—C20—H20B	109.6
N2—C9—C8	110.34 (8)	C24—C20—H20B	109.6
C13—C9—C8	110.24 (8)	H20A—C20—H20B	108.1
N2—C9—H9A	111.1	C16—C21—H21A	109.5
C13—C9—H9A	111.1	C16—C21—H21B	109.5
C8—C9—H9A	111.1	H21A—C21—H21B	109.5
N1—C10—C14	124.23 (9)	C16—C21—H21C	109.5
N1—C10—N2	114.20 (9)	H21A—C21—H21C	109.5
C14—C10—N2	121.57 (9)	H21B—C21—H21C	109.5
C17—C11—C16	118.70 (9)	O2—C22—H22A	109.5
C17—C11—H11A	120.7	O2—C22—H22B	109.5
C16—C11—H11A	120.7	H22A—C22—H22B	109.5
O5—C12—N2	127.50 (10)	O2—C22—H22C	109.5
O5—C12—C15	126.81 (9)	H22A—C22—H22C	109.5
N2—C12—C15	105.66 (8)	H22B—C22—H22C	109.5
C15—C13—C18	129.15 (9)	O4—C23—H23A	109.5
C15—C13—C9	109.65 (9)	O4—C23—H23B	109.5
C18—C13—C9	121.10 (9)	H23A—C23—H23B	109.5
C16—C14—C10	118.22 (10)	O4—C23—H23C	109.5
C16—C14—H14A	120.9	H23A—C23—H23C	109.5
C10—C14—H14A	120.9	H23B—C23—H23C	109.5
O1—C15—C13	127.97 (9)	C20—C24—H24A	109.5
O1—C15—C12	122.13 (9)	C20—C24—H24B	109.5
C13—C15—C12	109.62 (9)	H24A—C24—H24B	109.5
C14—C16—C11	118.34 (10)	C20—C24—H24C	109.5
C14—C16—C21	120.49 (10)	H24A—C24—H24C	109.5
C11—C16—C21	121.16 (10)	H24B—C24—H24C	109.5
N1—C17—C11	124.02 (10)		
			
C22—O2—C8—O3	−11.71 (15)	C20—O1—C15—C13	−130.46 (11)
C22—O2—C8—C9	166.06 (8)	C20—O1—C15—C12	56.26 (13)
C12—N2—C9—C13	−1.63 (11)	C18—C13—C15—O1	7.41 (18)
C10—N2—C9—C13	176.88 (8)	C9—C13—C15—O1	−176.23 (10)
C12—N2—C9—C8	115.93 (9)	C18—C13—C15—C12	−178.63 (10)
C10—N2—C9—C8	−65.56 (11)	C9—C13—C15—C12	−2.27 (11)
O3—C8—C9—N2	−40.90 (13)	O5—C12—C15—O1	−2.48 (16)
O2—C8—C9—N2	141.24 (8)	N2—C12—C15—O1	175.60 (9)
O3—C8—C9—C13	71.98 (12)	O5—C12—C15—C13	−176.85 (10)
O2—C8—C9—C13	−105.87 (9)	N2—C12—C15—C13	1.22 (11)
C17—N1—C10—C14	1.13 (15)	C10—C14—C16—C11	0.02 (15)
C17—N1—C10—N2	−179.48 (8)	C10—C14—C16—C21	178.63 (9)
C12—N2—C10—N1	171.31 (9)	C17—C11—C16—C14	1.30 (15)
C9—N2—C10—N1	−6.97 (13)	C17—C11—C16—C21	−177.30 (10)
C12—N2—C10—C14	−9.29 (16)	C10—N1—C17—C11	0.34 (15)
C9—N2—C10—C14	172.43 (9)	C16—C11—C17—N1	−1.56 (16)
C10—N2—C12—O5	0.04 (17)	C23—O4—C18—O6'	−12.9 (5)
C9—N2—C12—O5	178.45 (10)	C23—O4—C18—O6	9.6 (5)
C10—N2—C12—C15	−178.01 (9)	C23—O4—C18—C13	177.55 (10)
C9—N2—C12—C15	0.39 (11)	C15—C13—C18—O6'	173.7 (5)
N2—C9—C13—C15	2.39 (11)	C9—C13—C18—O6'	−2.3 (5)
C8—C9—C13—C15	−115.24 (9)	C15—C13—C18—O6	151.4 (5)
N2—C9—C13—C18	179.09 (9)	C9—C13—C18—O6	−24.6 (5)
C8—C9—C13—C18	61.45 (12)	C15—C13—C18—O4	−16.81 (16)
N1—C10—C14—C16	−1.31 (16)	C9—C13—C18—O4	167.21 (9)
N2—C10—C14—C16	179.34 (9)	C15—O1—C20—C24	66.34 (12)
